# CODE BLUE-19: Proposal to Mitigate COVID-19 Transmission in the Emergency Department for Out-of-hospital Cardiac Arrest

**DOI:** 10.5811/westjem.2020.7.48436

**Published:** 2020-09-24

**Authors:** David Nguyen, Nima Sarani, Kenneth D. Marshall, Chad M. Cannon, Ryan C. Jacobsen, Andrew Pirotte, Christine Pittenger, Edric K. Wong, Nicholas P. Dodson, Maria LaCapra, Kelly Howe

**Affiliations:** * Kansas University Medical Center, Department of Emergency Medicine, Kansas City, Kansas; † Kansas University Medical Center, Department of Pharmacy, Kansas City, Kansas

## Abstract

Resuscitation of cardiac arrest in coronavirus disease 2019 (COVID-19) patients places the healthcare staff at higher risk of exposure to severe acute respiratory syndrome coronavirus 2 (SARS-CoV-2). Unfortunately, COVID-19 status is unknown in most patients presenting to the emergency department (ED), and therefore special attention must be given to protect the healthcare staff along with the other patients. This is particularly true for out-of-hospital cardiac arrest patients who are transported to the ED. Based on the current data available on transmissibility of SARS-CoV-2, we have proposed a protocolized approach to out-of-hospital cardiac arrests to limit risk of transmission.

## BACKGROUND

It has been recognized that cardiopulmonary resuscitation (CPR) is an aerosol-generating procedure (AGP).^1^ In fact, there is evidence of transmission of severe acute respiratory syndrome coronavirus and Middle East respiratory syndrome to healthcare workers involved in CPR despite wearing proper airborne personal protective equipment (PPE).^2^,^3^ Considering the growing number of severe acute respiratory syndrome coronavirus 2 (SARS-CoV-2) infections in the United States and around the world, many healthcare workers will provide direct care to patients suffering cardiac arrest who are suspected of having coronavirus disease 2019 (COVID-19). Careful planning and thorough preparation are required to deliver quality resuscitation while protecting staff and other patients from potential exposure to the virus. In addition, mindful deployment of available resources must be weighed against resuscitative efforts and patient outcomes.^4^

Given that the overall survival to discharge of out-of-hospital cardiac arrest (OHCA) is approximately 8.8%, the benefits of resuscitation now must be weighed against transmission risk to providers.^5^ The emergency physician should assess the likelihood of neurologically intact survival in each OHCA and decide whether to continue resuscitation prior to the patient entering the emergency department (ED).^6^ Placement of an ultrasound machine in the ambulance bay prior to patient arrival to quickly visualize cardiac activity may assist with decision-making.^7^ With this in mind, the following is a proposed integrative protocol for OHCA during the COVID-19 pandemic.

## TRANSMISSION

With the emergence of the SARS-CoV-2 and its related disease process (COVID-19), the world faces a pandemic that has drawn comparison to that of the Spanish influenza outbreak of 1918.^8^ At the time of this writing, over 13.3 million cases have been confirmed and over 580,300 deaths have been associated with COVID-19. Of these confirmed cases, over 3.4 million were identified in the United States.^9^

Viral transmission has been identified by both respiratory droplet and surface contact. Mitigation of transmission has become a key strategy. Across the world, measures such as stay-at-home orders, school closures, travel bans, self-quarantine, and the implementation of physical distancing have been recommended.^10^ The workforce has made rapid, large-scale adaptations to reduce disease transmission, including a major shift to working from home and conducting business remotely with online applications.

In contrast, for first responders and front-line healthcare workers, contact with confirmed and suspected COVID-19 patients cannot be avoided, and the risk must instead be managed and mitigated. Moreover, many of these personnel will be involved in or exposed to AGPs. Examples of these interventions include endotracheal intubation, open suctioning, manual ventilation before intubation, and CPR.^11^ All of these procedures may be involved in the resuscitation of a patient in cardiac arrest. Noting that cardiac injury and cardiac arrest are among observed complications of COVID-19, it is critical to reconsider pre-pandemic approaches to cardiac arrest patients.^12^ Our institution, in partnership with the region’s emergency medical services (EMS) agencies, has developed a protocolized approach to managing the EMS-to-ED care transition for OHCA.

Several key assumptions guide this strategy. First, under pandemic conditions, it is important to devise protocols that assume risk of COVID-19 infection in all OHCA. Furthermore, if the cause of cardiac arrest is due to COVID-19 infection, then the patient is likely to exhibit persistent oropharyngeal viral shedding.^13^ This shedding creates a high-risk environment for resuscitation. Planning and preparation for these patient encounters and acknowledging available resources is vital to optimizing care while protecting healthcare workers and other patients from transmission.

## PROTOCOLIZED APPROACH TO SUSPECTED COVID-19

This protocol was developed at a tertiary, academic hospital within a large metropolitan area served by multiple EMS agencies, each with different capabilities, equipment, and protocols. The intent was to minimize dispersion of aerosolized viral particles throughout the ED, while maintaining optimal personnel, equipment, medications, and communication to facilitate high-quality resuscitation throughout care transfer. To achieve these goals, the following are required:

- Ensure that necessary staff have donned appropriate personal protective equipment (PPE) before they assume care of the patient
- Reduce the number of providers in the room to the fewest possible while still allowing optimal resuscitation
- Encourage use of negative pressure rooms, or identify resuscitation rooms with portable, high efficiency particulate air (HEPA) filter units
- Reduce the time during which the patient is receiving CPR in the ED but outside the negative pressure environment by minimizing travel distance from the ambulance bay to the negative pressure room.
- Minimize staff ingress and egress from the resuscitation room
- Reduce likelihood of an “open” airway (ie, an airway without a viral filter in place on an endotracheal tube or supraglottic device) during chest compressions inside the ED
- Mitigate the risk of aerosolized viral particle dispersion outside the negative pressure room.

**Figure 1:**
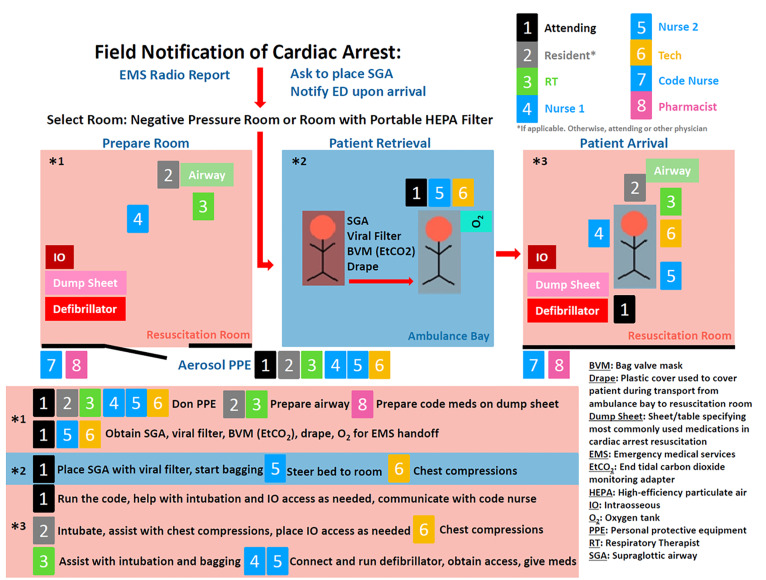
Illustrative diagram of code team and responsibilities to mitigate COVID-19 transmission.

To achieve these goals, the protocol recommends specific physical placement of equipment and personnel both prior to and during a resuscitation. The code team for this protocol includes an attending physician, a senior emergency medicine resident physician, three nurses including the code narrator (code nurse), a respiratory therapist (RT), an ED tech (code tech), and an ED pharmacist. To minimize infection risk, the code team consists of two teams: one team inside the room, and the second team outside (Figure 1). Use of an automated compression device to replace the code tech, if available, further reduces the number of the in-room team members.

**Figure 2:**
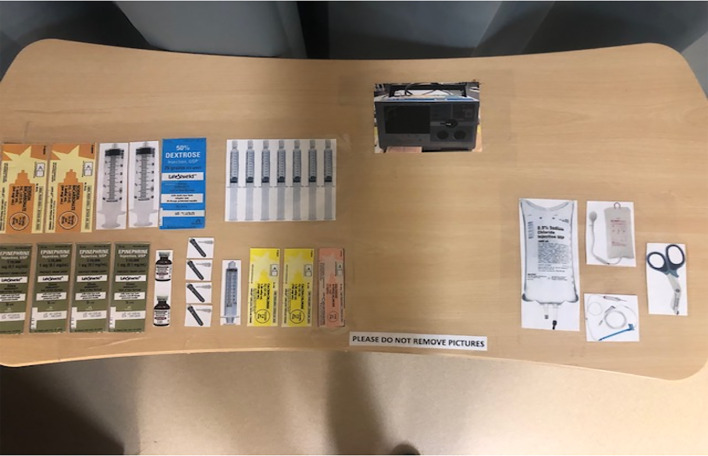
“Dump sheet” for cardiac arrest COVID-19 response. Medications most commonly used in cardiac arrest resuscitation and a defibrillator are pre-positioned by the code team on a sheet. Its purpose is to avoid contaminating the crash cart.

**Figure 3:**
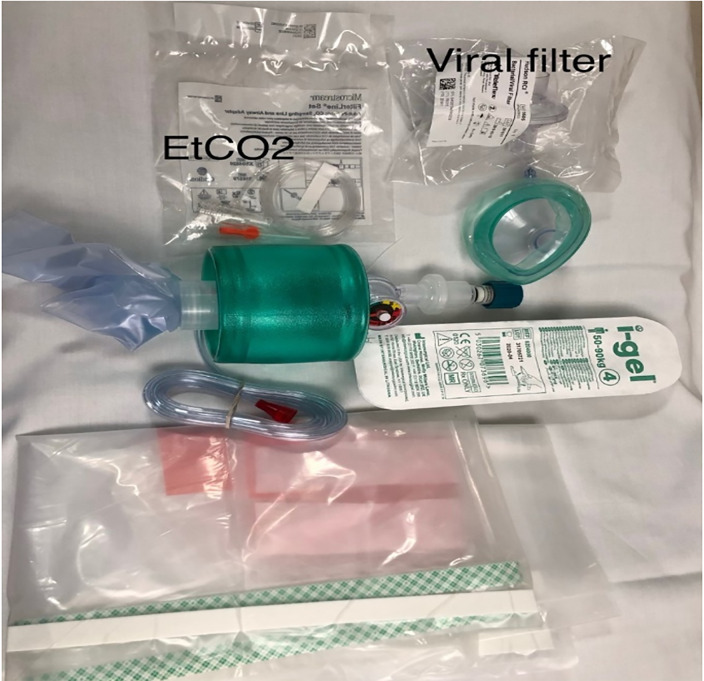
Cardiac arrest aerosol mitigation bag for cardiac arrest COVID-19 response. This bag includes a supraglottic airway device, bag valve mask, viral filter, end tidal carbon dioxide monitoring adapter, and a plastic drape used to cover patient.

**Figure 4:**
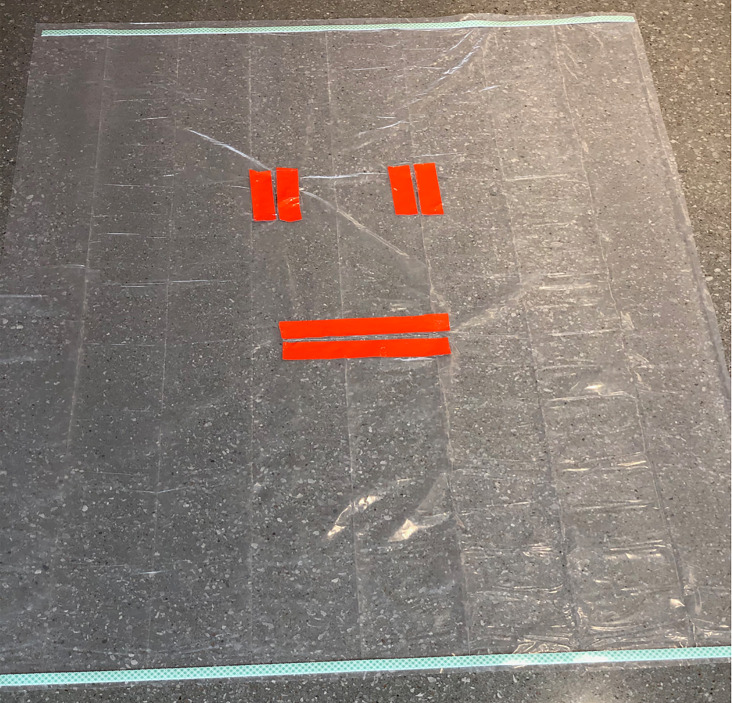
Plastic drape for cardiac arrest COVID-19 response. Used to cover patient during transport from ambulance bay to the resuscitation room. The drape has two vertical slits on top allowing the operator to access the airway and one horizontal slit below allowing for chest compressions or for further access as needed without having to remove the drape.

PPE is pre-staged outside of designated negative pressure rooms to facilitate use and availability. Medications most commonly used in cardiac arrest resuscitation and a defibrillator are pre-positioned by the code team on a sheet (referred to as the dump sheet, Figure 2) while the code cart stays with the out-of-room team to minimize contamination, ingress, and egress. To further minimize ingress and egress, multiple modes of communication are brought into the room, including portable two-way radios, pre-printed cards with common terminology, and dry-erase white boards and markers. Moreover, to reduce the likelihood of an open airway and mitigate viral particle dispersion, a pre-staged cardiac arrest aerosol mitigation bag is positioned on the route to the ambulance bay, containing a bag-valve-mask (BVM), a supraglottic airway device (SGA), a viral filter, lubricating jelly, and a clear plastic drape (Figures 3–4). In the event that an SGA has not been placed in the OHCA by EMS personnel, the attending physician places the SGA while in the ambulance bay. The use of a viral filter on the SGA and drape placement over the patient’s entire body serve to mitigate aerosolized viral particle dispersion while outside of the resuscitation rooms. This protocol reflects the availability of certain resources and personnel, such as negative pressure rooms, portable HEPA filter units, and resident physician providers. This protocol can be modified based on available resources.

## EXAMPLE PROTOCOL

### EMS report

- EMS contacts ED to report cardiac arrest with ongoing CPR.
- If airway is not already secured by advanced airway such as endotracheal tube or SGA, ED personnel requests EMS personnel to place an SGA and meet the ED team in the ambulance bay for patient handoff.

### CODE Team and PPE

- Nursing supervisor identifies and notifies both the in-room team (an attending physician, a senior emergency medicine resident physician, two in-room nurses, an RT, and a code tech) and the out-of-room support team (a code nurse and an ED pharmacist) at the time of EMS call-in.
- Members of the in-room team don airborne PPE prior to patient arrival and remain inside the resuscitation room while the code nurse and the ED pharmacist remain outside of the room for the entire duration of the code. Communication between the attending physician who remains inside the room wearing airborne PPE and the code nurse outside of the room is through multiple means, including a hand-held, portable two-way radio, dry-erase white boards and markers, and pre-printed cards with common terminology. For example, the attending physician holds the in-room radio and speaks directly to the code nurse who is holding the out-of-room radio.

### Prior to Arrival

- The in-room team has two separate objectives prior to patient arrival. A handoff sub-unit consisting of the attending physician, the code tech, and one of the in-room nurses dons airborne PPE upon EMS report, obtains the cardiac arrest aerosol mitigation bag (Figure 2) and a portable radio, and pre-stages the resuscitation room stretcher equipped with an oxygen tank in the ambulance bay to meet EMS.
- Other personnel of the in-room team, the resident physician and RT, don airborne PPE and prepare for airway management, including preparing the video laryngoscope, ensuring the supplies needed to intubate are present, and confirming that the ventilator is optimally configured for viral filtration.
- Secondary in-room nurse works with the ED pharmacist to prepare code medications and ensure defibrillator and intraosseous availability. The code cart remains outside of the room while commonly administered resuscitation medication for a typical code duration is placed inside the resuscitation room (Figure 1).
- The code nurse remains outside of the room and communicates with the attending physician through a portable two-way radio.

### On Arrival to the Ambulance Bay

- The patient is transferred from the EMS stretcher to the ED stretcher. The attending physician places an SGA (if not already in place) and connects the viral filter between the SGA and BVM. EMS report is given during this transition.
- The attending physician provides ventilation by bagging the patient and the ED tech provides compressions. The nurse pushes the stretcher to the resuscitation room. Ideally the plastic drape is on the patient during transport from ambulance bay to the room (Figure 3).

### Patient in Room

- As the patient enters the room, the door closes to activate the negative airflow. The attending gives a short report over the radio to the code nurse outside of the room regarding the events prior to arrival including downtime, rhythm, and shocks and medications that have been delivered as reported by EMS. The code nurse assumes timekeeping of code events.
- The code tech continues compressions until the first rhythm and pulse check.
- Meanwhile, the primary nurse attaches the patient to the monitor and defibrillator.
- The resident physician intubates the patient during first pulse check*. If a resident physician is not available, then the attending physician performs the intubation.
- The secondary nurse establishes intravenous (IV) access if not already established by EMS and administers medications or draws blood as needed.
- The attending physician leads the code and communicates all events in the room to the code nurse outside. The attending places an IO if required.
- After intubation, the resident physician participates in providing chest compressions and rotates with the ED tech and nurse for the duration of the code.

*Ordinarily, an SGA is sufficient for ventilation during CPR. However, under current pandemic conditions, the assumption is that a cuffed tube in the trachea is preferred to decrease expiratory leak and further mitigate contamination.^14^

### Outside the Room

- The code nurse radios to the team when it is time for rhythm checks, pulse checks, and medication administration.
- The ED pharmacist outside the room assists in making suggestions for medication and fluid administration related to cardiac arrest resuscitation guidelines and assists the code nurse in documenting events. Furthermore, the pharmacist procures and prepares other necessary medications that are not in the room.

### Achieving Return of Spontaneous Circulation (ROSC)

- The attending physician gives orders for an electrocardiogram, additional labs, arterial line placement, continuous IV fluids and medications, etc. through the radio. The out-of-room team prepares and delivers these items.
- The team in the room stays in airborne precautions PPE for 30 minutes post-AGP procedure (intubation) if providing care inside the room.^15^

### Patient Death

- The attending physician announces time of death to both teams; the out-of-room nurse documents this finding in the chart.
- The patient remains in the negative pressure room for 30 minutes post-AGP to reduce any aerosols. The patient is then double-bagged, with “contaminated” and “biohazard” stickers placed on the outside of the bag.

## CHALLENGES

This protocol is intended to minimize risk of healthcare exposure to SARS-CoV-2 while delivering quality resuscitation for cardiac arrest. To date, there is limited evidence to estimate COVID-19 transmission during care for cardiac arrest. Development of practice protocols requires a balanced approach: weighing an unknown transmission risk against known risk to the patient from treatment delays.^16^

### Several Additional Notes

The protocol reflects certain institutional-dependent aspects that may require modification to generalize. For example, certain team members such as resident physicians and pharmacists may not be available to fulfill these roles at other institutions.

Regarding airway management, available resources and provider experience should guide intervention. For example, in a setting where the clinician is inexperienced in airway management and a video laryngoscope is not available, an SGA may be preferred.

The protocol aims to minimize the number of individuals involved with the resuscitation. Given this recommendation, there is great utility in an automated compression device, acknowledging the financial resources required for such a device.

Additionally, early iterations of this protocol used white boards and preprinted code-communication sheets for communication. These efforts were subsequently deemphasized in favor of radio or phone communication, which proved to be audible despite the background noise and allowed for an easier conversation between the in-room and out-of-room teams. However, these options remain available as an alternative and backup form of communication. As an example, laminated cards with events such as rhythm check, items such as medications, and common questions were deployed in the negative pressure rooms.

Another limitation of this protocol is who is using the communication devices. Designed for an academic institution, the protocol designates the attending hold the radio in room, whereas in non-academic settings the physician may or may not have the capacity to act as the communications liaison and may have to delegate that role to another individual. All communication devices should be disinfected with EPA-approved, low-level disinfection (LLD) between each patient encounter, in accordance with local institutional biomedical equipment cleaning guidelines.^17^

Observations during arrests suggest the plastic drape has potential to interfere with resuscitation and may need to be removed after arrival into the resuscitation room. The design of the drape consists of two vertical slits on top for airway access and a large horizontal slit on the bottom for direct chest compressions. Despite this unique design, the drape may be associated with technical difficulties, including difficulty in manipulating airway equipment, and drape movement during compressions.

Point-of-care ultrasound (POCUS) can provide valuable information during cardiac arrest, including identification of interventions outside of the standard Advanced Cardiac Life Support algorithm. Importantly, it has been demonstrated that patients in asystole with no cardiac activity on POCUS have low chance of survival to hospital discharge. In such scenarios, prolonging CPR is likely unavailing, and under the current pandemic may further expose the healthcare worker to SARS-CoV-2. Potentially, an ultrasound check in the ambulance bay could allow calling the code in appropriate circumstances prior to bringing patient into the ED. If POCUS is used, a designated location for the ultrasound machine in the ambulance bay could reinforce its use. The ultrasound machine should be disinfected with LLD after every use, in accordance with local institutional biomedical equipment cleaning guidelines.^18^

Lastly, advanced discussions with EMS agencies regarding expectations are vital to implementing this protocol. A challenge to these discussions is the multitude of EMS agencies that may respond to a facility, and the potential need to coordinate with each one independently. Even in jurisdictions with minimal out-of-hospital practice variation due to statewide EMS protocols, coordination with EMS agencies is still necessary in order to anticipate and adjust for specific scenarios such as severe weather conditions for hospitals without ambulance bays.

## CONCLUSION

Cardiac arrest management is always challenging. Cardiac arrest management during an evolving pandemic poses additional challenges. These recommendations are intended to assist our local, regional, national, and global medical colleagues in the care of cardiac arrest during the pandemic. This protocol is specific to our institution but may be modified to meet the needs of others. All efforts aim to safeguard providers while maintaining high quality of care for our patients.
